# Rarity-Weighted Change Score: An Integrative, Distribution-Aware Measure of Pre-Post Outcomes in Psychotherapy Research

**DOI:** 10.7759/cureus.106490

**Published:** 2026-04-05

**Authors:** Bangalore Roopesh

**Affiliations:** 1 Clinical Psychology, National Institute of Mental Health and Neurosciences, Bengaluru, IND

**Keywords:** outcome measurement, patient outcome research, pre-post therapy outcome measurement, psychotherapy research, rarity weighted change score

## Abstract

In psychotherapy outcome research, the gold standard to measure the effectiveness of any psychotherapy is to determine the extent of change or difference in the outcome variable at post-intervention compared to pre-intervention assessment. Depending on the type of distribution, analysis of the change score is generally carried out using paired t tests and/or repeated measures ANOVA for within-group analysis. If there is more than one group, such as intervention and control groups, then usually a two-way repeated-measures ANOVA or mixed-effects model is used. These standard analysis procedures are followed with the assumption that all change scores are equivalent in terms of difficulty and magnitude. However, a simple change score does not characterize the complexity involved in the outcome of an intervention. A few methods - such as the Reliable Change Index, calculating proportional change, and calculating percentile norms - capture some of the complexities involved but do not include most of them, such as accounting for baseline difficulty, distribution of pre-post scores, and how common or rare a given improvement is. This methodological gap in evaluating psychotherapy outcome research necessitated the development of the Rarity-Weighted Change Score (RWCS) approach. This integrative approach incorporates percentile-based rarity of change, baseline difficulty, and outcome extremity adjustment into a single composite score. This approach yields comprehensive, standardized, distribution-aware scores that are suitable for both simple descriptive and complex inferential statistical analysis. This approach is intuitive, easier to calculate, and adds value to the psychotherapy outcome research.

## Introduction

In psychotherapy research, improvement can signify two broad aspects: (1) increase from lower to higher scores in domains such as well-being, awareness, emotional regulation, mindfulness, resilience, quality of life, skills and/or functionality, and (2) decrease in symptom scores from higher to lower in conditions, such as anxiety severity, depression severity, emotional dysregulation, interpersonal conflict, substance use and/or stress levels. Depending on the design and the objectives of the study, traditional approaches have used different metrics and statistical analyses [1,2,3,4]. One of the earlier methods used was comparing the pre- and post-intervention scores of the outcome variable using a paired t-test or repeated measures ANOVA. In some instances, analysis of covariance (ANCOVA) or repeated measures ANCOVA with pre-intervention raw scores as the covariate was also used. However, these statistical measures may provide limited insight into individual variability and do not account for clinical significance [5,6].

Raw change score or change score

To overcome some of the difficulties of the traditional approaches, researchers have used the difference in the raw scores between pre- and post-intervention, often termed as the ‘raw change scores’ or simply the ‘change scores’, for various analyses, such as to compare the treatment effects between the groups [7], and/or as dependent variables in regression models [8,9,10]. Further, as it is a single (raw change) score, as opposed to two (pre- and post-intervention) scores, it was considered more appropriate for group comparison, regression, or any other statistical techniques [11].

However, using raw change scores creates several conceptual and statistical limitations. They are (a) raw change scores are inherently biased with respect to baseline scores. For example, if the outcome variable is resilience, and if a participant starts with a lower baseline score at pre-intervention, they have more room for improvement in the post-intervention assessment when compared to the person starting at the higher baseline score; (b) raw change scores alone do not do justice to the magnitude/difficulty of change, or as to where on the scale the change has occurred. The similar extent of change score near the ceiling of person A is different compared to the same change score near the floor; (c) raw change score does not account for how everyone has changed relative to each other, i.e., the distribution of scores; and (d) raw change scores are not standardized scores.

Considering these issues in psychotherapy and clinical outcome studies, researchers have come up with various approaches. Some of them are Reliable Change Index (RCI) [12], Percent/Proportional change method [13], Percentile Norms for Outcome method [14], and Growth Curve Modelling [14]. Among all, RCI is the most widely used approach, as it evaluates whether an individual’s pre-post score difference exceeds the amount of change expected due to measurement unreliability alone [12]. On the other hand, some researchers use proportional or percent change (instead of the raw change score) from baseline, which addresses baseline dependence by expressing improvement relative to initial severity [13]. Along similar lines, some researchers interpret outcome data using normative and percentile ranks (e.g., percentile ranks/norms for baseline and post-intervention scores) [13,14]. Another approach that takes into account both the 'pre' and 'post' values is Growth Curve Modelling [15].

Given the limitations in various approaches, the article proposes an integrative approach combining distribution or percentile-based rarity of change, depending on the minimum and maximum obtainable score of the scale, adjusting for baseline (pre-intervention) difficulty, and for outcome (post-intervention) extremity, as well as accounting for both improvement and deterioration, into a unified standardized measurement of Rarity-Weighted Change Score.

## Technical report

Methods

The Rarity-Weighted Change Score method follows six steps to arrive at the final value. The entire procedure is depicted in Table 1 and is detailed below. In addition, a sample group is depicted in Table 2 to help understand the steps.

**Table 1:** Steps to arrive at the RWCS In the Excel formulae given in this table, the capital letters represent the columns in the Excel worksheet, number 2 represents row 2, and the number 6 represents row 6. This Excel formula applies to the group of five subjects (as shown in Table 2). This can be changed depending on the number of participants in the group. Excel by Microsoft Corporation, Redmond, USA. RWCS: Rarity-Weighted Change Score; RWC-SS: Rarity-Weighted Change – Scale Score; RWC-Z = Rarity-Weighted Change – Z Score; MinRWCS: Minimum Rarity-Weighted Change Score; MaxRWCS: Maximum Rarity-Weighted Change Score.

Step		Conceptual formula	Excel formula
1	a	Change score = Post – Pre-intervention score	= C2–B2
	b	Change score = Pre – Post-intervention score	= B2–C2
2		Change Rarity = Percentile Rank	=PERCENTRANK.INC($D$2:$D$6, D2)
3		Baseline/Pre Difficulty = Percentile Rank	=PERCENTRANK.INC($B$2:$B$6, B2)
4		Outcome/Post extremity = Percentile Rank	=PERCENTRANK.INC($C$2:$C$6, C2)
5		RWCS = Change rarity + Baseline difficulty + Outcome extremity	=E2+F2+G2
6	a	RWC_SS = ((RWCS − MinRWCS) / (MaxRWCS − MinRWCS)) × 100	=((H2-MIN($H$2:$H$6))/(MAX($H$2:$H$6)-MIN($H$2:$H$6)))*100
	b	RWC-Z = (RWCS − Mean RWCS) / SD	=(H2-AVERAGE($H$2:$H$6))/STDEV.S($H$2:$H$6)

**Table 2:** Simulation of all the steps to calculate RWCS in a sample of five subjects The first row lists the letters B to J, the default alphabet columns in the Excel sheet where the respective values will be entered. Pre- and baseline are used interchangeably. Excel by Microsoft Corporation, Redmond, USA. Pre: pre-intervention assessment score; post: post-intervention assessment score; RC: raw change; CR: change rarity in percentiles; BD: baseline difficulty (pre-intervention score) in percentiles; OE: outcome extremity (post-intervention score) in percentiles; RWCS: Rarity Weighted Change Score; RWC-SS: Rarity-Weighted Change – Scale Score; RWC-Z = Rarity-Weighted Change – Z Score.

Sample group									
B	C	D	E	F	G	H	I	J	← Excel column
Pre	Post	RC	CR	BD	OE	RWCS	RWC-SS	RWC-Z	Possible interpretation
		1	2	3	4	5	6a	6b	← Calculation Steps
60	80	+20	1.00	1.00	1.00	3.00	100	+1.36	Extremely strong composite outcome
50	60	+10	0.75	0.50	0.75	2.00	66.7	+0.48	Above-average composite outcome
50	50	0	0.25	0.50	0.50	1.25	41.7	-0.18	Slightly below composite outcome
40	45	+5	0.50	0.25	0.25	1.00	33.3	-0.39	Below-average composite outcome
35	30	-5	0.00	0.00	0.00	0.00	0	-1.27	Weakest composite outcome

​​​​​​​​​​​​​​*Step 1: Raw Change (RC)*

Calculate the raw change score to capture the basic improvement. Improvements in psychotherapy are determined based on the type of variable used as the outcome measure. This can be categorized into two types. As mentioned earlier, these are (a) ‘improvement scores’, such as awareness, emotional regulation, or quality of life; and (b) ‘symptom scores’, such as anxiety-depression severity, stress, or substance use.

Step 1a: If the outcome variable uses improvement variables, the raw change score is arrived at by subtracting the pre-intervention score from the post-intervention score. Here, the raw change scores can either be positive values indicating improvement or negative values indicating deterioration after treatment.

Step 1b: If the outcome variable assesses symptoms, the raw change score is arrived at by subtracting the post-intervention assessment score from the pre-intervention assessment score. Here, as in the improvement variable, the raw change scores can either be positive values indicating improvement or negative values indicating deterioration after treatment.

This is the only change in the calculation that the researchers are expected to do, depending on the type of outcome variable, i.e., improvement or symptom scores. All other steps (from Step 2 to Step 6b) will be the same, irrespective of whether the outcome variable is an improvement score or a symptom score.

Step 2: Change Rarity (CR)

In this step, all the change scores are converted into percentile ranks within the particular group. Thus, the percentile represents ‘how rare the improvement is relative to the group distribution’. This step is direction sensitive, where, along with the extent of the score, negative scores have a lower percentile rank compared to positive scores. In other words, improvements receive a higher percentile compared to deterioration, and higher scores receive a higher percentile compared to lower scores. In terms of interpretation, higher percentiles indicate greater improvements, and vice versa. The change value sorted for the sample group is -5, 0, +5, +10, +20 (Table 2).

Step 3: Baseline Difficulty (BC)

Next, baseline scores are also converted into percentile ranks. This step accounts for ‘how difficult improvement was, given the participant’s starting point’. Participants who begin with higher baseline scores generally have less room to improve because they are closer to the maximum possible score. Hence, the higher the baseline (pre) scores receive, the higher the weight (percentile score).

Step 4: Outcome Extremity (OE)

This step captures the strength of the final outcome of the intervention. Here, post-intervention scores are converted into percentile scores. Participants who end up improving more after the treatment receive higher credit in terms of higher percentiles.

Step 5: Rarity-Weighted Change Score (RWCS)

In this step, the three percentile-based components (change rarity, pre/baseline difficulty, and post-outcome extremity) that are obtained in Steps 2, 3, and 4 are combined. As in each of these three components, the percentiles range from 0 to 1, and the RWCS ranges from 0 to 3. Higher scores indicate improvement that is rare relative to the group, achieved despite baseline difficulty, and associated with strong final performance.

Step 6a: Rarity-Weighted Change - Scaled Score (RWC-SS)

To improve interpretability, the RWCS is rescaled to a 0-100 index. Given this, a score closer to 0 indicates the weakest composite outcome, closer to 50 indicates the mid-range composite outcome, and closer to 100 indicates the strongest composite outcome of intervention. This scaling mainly helps to easily visualize and interpret the data. However, RWC-SS cannot be used for inferential statistics, as it does not preserve the original statistical distribution.

Step 6b: Rarity-Weighted Change - Z** **Score (RWC - Z)

To statistically compare the resultant values across the groups, RWCS (Step 5) needs to be converted to the Z scores (RWC-Z), which indicate how far a participant’s composite outcome lies from the group average. A score of +1.0 indicates one standard deviation above average composite outcome, 0 indicates average composite outcome, and -1.0 indicates below average composite outcome. Here, one has to note that RWC-Z is derived from RWCS (Step 5 outcome) and not from RWC-SS (Step 6a outcome).

**Figure 1:**
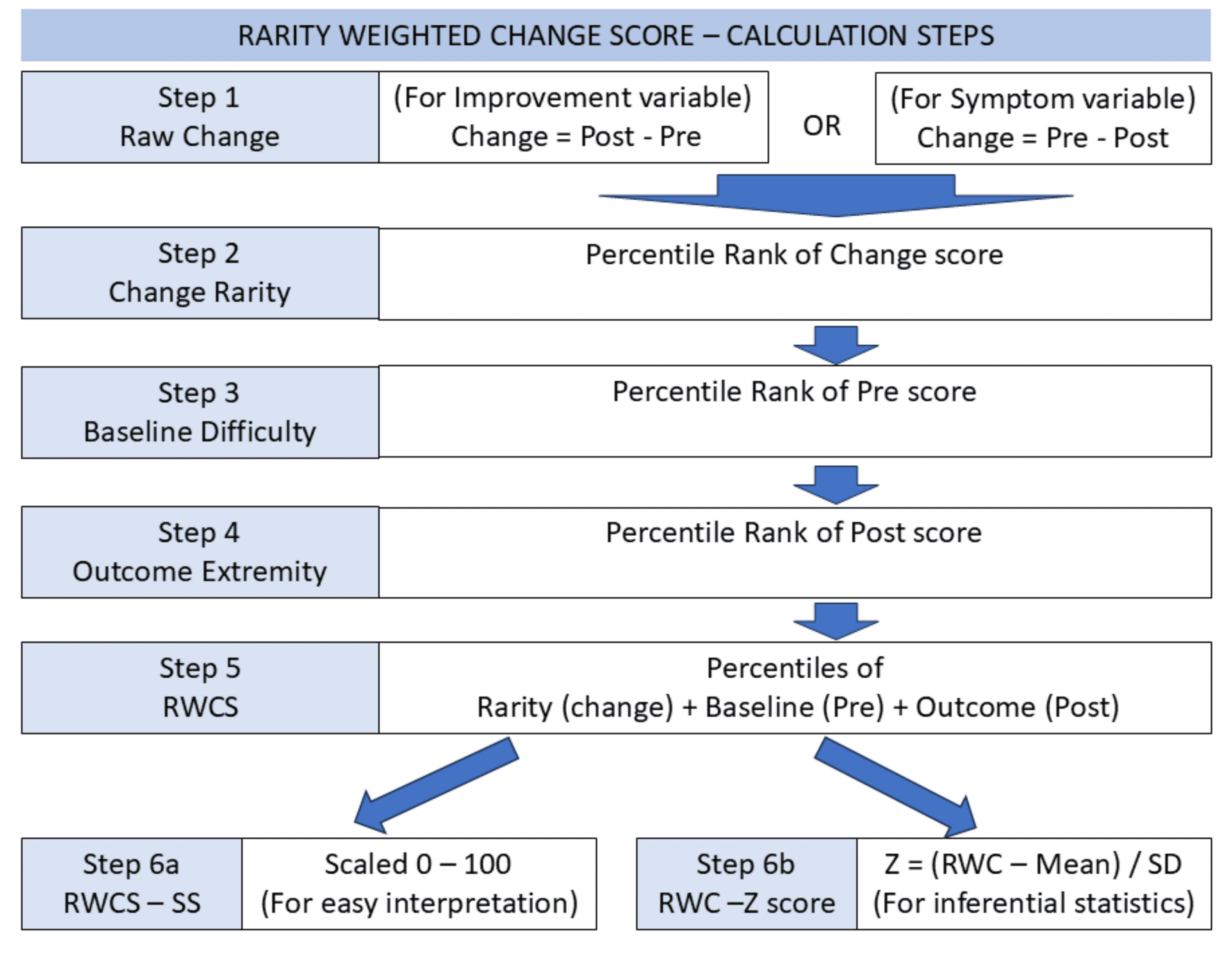
Steps to arrive at the RWCS RWCS: Rarity-Weighted Change Score; RWC-SS: Rarity-Weighted Change – Scale Score; RWC-Z = Rarity-Weighted Change – Z Score. Figure created by the author using Microsoft PowerPoint software (Microsoft Corporation, Redmond, USA).

All the procedural steps have been presented in Table 1 and Figure 1. The simulation of the RWCS analysis for five subjects is given in Table 2. It should be noted that in Table 2, the pre- and post-assessment scores are the same, but the interpretation indicates a slightly below-average composite outcome. In terms of the score, there is no change in the outcome; in terms of the group, it can be interpreted as a below-average composite outcome. 

Stability of the intervention (follow-up) analysis

Researchers who are interested in whether the changes observed in post-intervention are maintained over time can use post-intervention and follow-up values, replacing the 'pre' and 'post' scores in the aforementioned steps.

## Discussion

Traditional approaches to studying the effectiveness of the psychological intervention did not want to restrict the analysis to just a dependent t-test or repeated measures ANCOVA in terms of statistics, and hence tried different approaches to measure both statistical and clinical changes [3,5]. One simple measure is to use the ‘raw change scores’ to compare between groups [7], or to use them as a dependent variable in regression models [8,10,11].

However, analysis using raw change scores, especially pre-post comparisons, is inherently biased by a person's pre-intervention score. Assuming that the outcome variable is well-being (score ranging from 20 to 100), where scores are expected to increase after the intervention; if participant S starts at a lower baseline score of 40 in pre- assessment, they have a chance of getting higher change score (100 - 40 = 60), compared to the participant T who starts with 65 (100 - 65 = 35). The opposite holds good if the outcome variable is a symptom score, anxiety, and/or depression severity. Similarly, raw scores do not consider where on the scale the change has occurred. For example, in the aforementioned scale, if both patients E and F obtain a raw change score of 10 points, but for patient E it is from the pre-post difference between 20 and 30, and for patient F it is from the pre-post difference of 75 to 85. Although the raw change scores are similar in both patients, it is clear that it is difficult to obtain the same near the higher extremes. In addition, the raw change score does not account for how everyone has changed relative to each other. In terms of groups, improvements are meaningful only when it is known how everyone has changed relative to each other. Distribution of scores within the group, both at pre-intervention assessment and at post-intervention assessment, plays a significant role in understanding the changes due to psychotherapy intervention. Closely related to this, raw change scores are not standardized scores, and hence, cannot be compared across different measures, different groups, and different time points.

Given the above limitations, the current proposed measure, RWCS, is an integrative, multi-level distribution-aware and standardized approach for evaluating changes after psychotherapy that tries to overcome the limitations, and in doing so, goes far beyond the simple raw change scores. 

Before this, several researchers have proposed various measures to go beyond the raw change scores. In this regard, the Reliability Change Index (RCI) has been extensively used in outcome measures, as it answers, 'did therapy really improve the symptoms', 'is the improvement larger than the measurement error', and 'which participant really changed', by classifying individuals as reliably improved, unchanged, or deteriorated [13,16,17]. However, despite its utility, RCI has certain limitations. It evaluates each person individually and ignores heterogeneity. In addition, it does not account for or adjust for extreme baseline scores (which tend to regress towards the mean) and how rare a given improvement is within the observed group, which can have implications for the outcome analysis and interpretation. Though the RWCS does not address the issue of the measurement error as in RCI, it does take into account the extreme scores in baseline, the rarity of the post-intervention score, and the distribution of all the metrics.

Similar to the RCI, another alternative measure - the proportional or percent change method [13] - also does not incorporate distributional rarity percentiles or extreme scores in the outcome variable at the post-intervention phase [13]. This limitation has been overcome in the RWCS approach. Further, another earlier measure of using only percentile ranks/norms for baseline and post-intervention scores [13,14] focuses on score interpretation rather than integrating the distribution of pre- and post-intervention scores, and how rare the difference (change) obtained into a unified composite change index, as done in the RWCS.

The Growth Curve Modelling (GCM) approach [15] shows promise as one of the better approaches to evaluate psychotherapy outcomes. Compared to RWCS, GCM provides trajectories, can handle more time points, and handles missing data, but is statistically very complex. In contrast, RWCS is relatively less sophisticated in terms of statistical sophistication, provides a single outcome score, and is very high in interpretability in terms of clinical aspects.

On the other hand, the most common argument one can put forth about the RWCS is that, instead of the lengthy procedure given herein, and as the ‘post-improvement score’ is already part of the ‘raw change score’, can one add the ‘pre score’ with the ‘raw change score’ to arrive at the ‘improvement index’? Though conceptually this appears correct, and the results are often used in ANCOVA, the aforementioned metric (e.g., improvement index = pre-intervention assessment score + raw change score) is just equivalent to the post-intervention assessment score. Table 3 clearly depicts this aspect, where the column ‘post’ and the column ‘pre+change’ have the same scores for the respective subjects. In this example, the common mistake in terms of interpretation is that one would favor participant M, because the improvement is larger (change score of 20), but clinically, participant N might be feeling better (post-intervention score of 40).

**Table 3:** An example of values that can be obtained by two subjects Pre: Pre-intervention; Post: Post-intervention

Participant	Pre	Post	Change	Pre + Change
M	10	30	20	30
N	30	40	10	40

Further, just ‘pre scores plus raw change’ score does not do justice for the outcome extremity or the post score, which (the latter) indicates how strong the final outcome is. In the procedure to calculate RWCS, all three scores (pre, post, and the raw change score) are treated independently to arrive at a weighted score that takes into account the difficulty at the start, the rarity of change, and the strength of the ending.

On the other hand, compared to just ‘pre-intervention + change score’, the procedure of RWCS uses percentiles. Percentiles solve three problems compared to the raw scores and/or raw change scores: (1) Raw scores depend on the scale, such as resilience, which can vary between 0 and 100, but emotional regulation might vary between 0 and 65. Given these differences, raw scores cannot be combined easily. However, percentiles convert everything into values from 0 to 1, and hence, all components become scale-free; (2) raw scores can be distorted by extreme values, but comparatively, percentiles are less sensitive to extreme values than raw scores; and (3) psychological measures are rarely normally distributed, but percentiles reduce reliance on distributional assumptions.

Another aspect one can question regarding the RWCS is that the change score is dependent on both pre- and post-intervention scores, and hence will lead to double-counting in terms of statistical procedure. Given this, within the current proposed procedure, a slight modification might appear to work on the surface level. It can be argued that, instead of ranking the post-intervention score alone, one can rank how high the person ended relative to others with a similar starting point. If one proceeds with this argument, the conceptual formula for this would be 'outcome advantage = post percentile minus pre percentile'. On the surface, this appears to solve the double-counting problem. However, it fails when a subject gets the same rank (which is possible in extreme scores) of 1 in both pre- and post-intervention, which would result in the score of 0 (i.e., ‘Post - Pre = outcome advantage’ is then ‘1-1 = 0'). This would disrupt the interpretation.

Further, there are a few things that need to be considered while using this procedure. RWCS is not a pure improvement/change measure alone, but it reflects a composite of improvement (change magnitude), baseline difficulty, and the final outcome position. Given this, it is better to use the term 'composite outcome' for the interpretation rather than 'improvement' for the outcome measure. Further, in cases where no change occurs, RWCS values reflect baseline and outcome positioning rather than improvement per se. Thus, non-zero scores under zero change should be interpreted as reflecting relative standing rather than change. 

Another important aspect is that percentile-based components in RWCS are computed relative to the sample distribution. As a result, values may not be directly comparable across groups if percentiles are derived separately within each group. However, for cross-group comparisons, percentile ranks can be computed using a pooled reference distribution across all groups to ensure a common scale. That is, the subject scores in both groups are pooled together to derive percentile ranks. Once the percentile ranks have been derived, the group analysis/comparison can be carried out with their respective percentiles. Given all the above alternative methods and their limitations, the current procedure from Steps 1 to 6b to arrive at RWCS is one of the methods to arrive at a composite index that is relatively simple to calculate and easy to interpret. However, in terms of psychotherapy pre-post improvement and group differences, repeated measures ANCOVA is still the go-to choice. In comparison, RWCS is more suited as an outcome variable when there is a need for prediction analysis for the improvement after psychotherapy, instead of simple change scores. That is, for answering questions such as 'What factors predict the improvement in any psychotherapy approach/technique?'.

Limitations

The RWCS approach can be one of the alternative approaches, but it does not address all aspects of improvement in psychotherapy research. It is intended primarily for descriptive comparison, clinical interpretation, or inferential statistical analyses. Though this is not a limitation in a strict sense, in RWCS, as the percentile ranks depend on the sample distribution, the results are group-dependent. As mentioned earlier, in scenarios with more than one group, the groups can be pooled together to arrive at percentile ranks, and these ranks can be used for inter-group comparison. In addition, the RWCS is a composite ordinal index rather than a linear metric. While percentile components are summed for interpretive convenience, the resulting score reflects relative standing across multiple dimensions rather than a true interval-scale measurement of change. Given this, in cases of tied values, equal scores receive identical percentile ranks based on their position within the sorted distribution. In small samples, or with Likert-type data, percentile ranks may become coarse and sensitive to ties. Future work should examine RWCS performance under varying sample sizes and distributions. In addition, given that percentile calculations can vary across software implementations, we specify the use of Excel’s PERCENTRANK.INC algorithm to ensure reproducibility. Further, RWCS can be treated as a descriptive composite index. As RWCS, especially the RWC_Z, is derived from dependent percentile ranks, its sampling distribution may deviate from normality, particularly in small samples or with discrete scales. Given this, inferential results based on RWC_Z should be interpreted cautiously, and/or non-parametric approaches might be considered to evaluate RWCS-based comparisons.

## Conclusions

On the whole, RWCS, including the RWC-Z score, integrates multiple dimensions of improvement into a single coherent metric. By considering the rarity of the change, the difficulty level of the starting point, and the strength of the final outcome, the procedure provides a more nuanced assessment than a simple raw change score. It should be noted that, when evaluating psychotherapy outcome, repeated measures ANCOVA (with baseline scores as covariates) and the Reliable Change Index are the valid and robust measures. However, when there is a need to use the improvement in the outcome variable for prediction analysis, these measures or the simple change scores do not do proper justice. In this scenario, RWCS provides an alternative way to evaluate any therapeutic improvement of an individual in the group, accounting for both individual performance and the distribution of scores within the group. As RWCS considers the distribution of the scores within the group, it is automatically considered norm-referenced. However, RWCS is dependent on group distribution, and if the need is to compare different groups, then the subjects of different groups need to be pooled together for percentile ranking, and then these ranks should be used for inter-group comparison. Further, as the final scores are added percentile ranks, the Z score derived may not be considered to be on an interval scale. Given these reasons, RWCS scores must be interpreted as composite scores.
